# HSP60 Regulates Monosodium Urate Crystal-Induced Inflammation by Activating the TLR4-NF-*κ*B-MyD88 Signaling Pathway and Disrupting Mitochondrial Function

**DOI:** 10.1155/2020/8706898

**Published:** 2020-12-31

**Authors:** Qiushi Huang, Wei Gao, Heng Mu, Ting Qin, Fan Long, Long Ren, Huan Tang, Jianping Liu, Mei Zeng

**Affiliations:** ^1^Institute of Rheumatology and Immunology, The Affiliated Hospital of North Sichuan Medical College, 63# Wenhua Road, Nanchong, 637000 Sichuan, Nanchong, China; ^2^Preclinical School of North Sichuan Medical College, 234# Fujiang Road, Nanchong, 637000 Sichuan, Nanchong, China; ^3^The Fifth People's Hospital of Nanchong City, 21#Bajiao Street, Nanchong 637100, Sichuan, Nanchong, China; ^4^Medical Imaging Key Laboratory of Sichuan Province, North Sichuan Medical College, 234# Fujiang Road, Nanchong 637000, Sichuan, Nanchong, China

## Abstract

Acute gout is an inflammatory response induced by monosodium urate (MSU) crystals. HSP60 is a highly conserved stress protein that acts as a cellular “danger” signal for immune reactions. In this study, we aimed to investigate the role and molecular mechanism of HSP60 in gout. HSP60 expression was detected in peripheral blood mononuclear cells (PBMCs) and plasma of gout patients. The effect and molecular mechanism of HSP60 in gout were studied in MSU crystals treatment macrophages and C57BL/6 mice. JC-1 probe and MitoSOX Red were used to measure the mitochondrial membrane potential (MMP) and mitochondrial reactive oxygen species (mtROS). HSP60 expression was significantly upregulated in the PBMCs and sera of patients with acute gout (AG) compared to those with intercritical gout (IG) or healthy controls (HCs). MSU crystals induced the expression and secretion of HSP60 in the macrophages. HSP60 knockdown or overexpression affects TLR4 and MyD88 expression, I*κ*B*α* degradation, and the nuclear localization of NF-*κ*B in MSU crystal-stimulated inflammation. Further, HSP60 facilitates MMP collapse and mtROS production and activates the NLRP3 inflammasome in MSU crystal-stimulated macrophages. In MSU crystal-induced arthritis mouse models pretreated with HSP60 vivo-morpholino, paw swelling, myeloperoxidase (MPO) activity, and inflammatory cell infiltration significantly decreased. Our study reveals that MSU crystal stimulates the expression of HSP60, which accelerates the TLR4-MyD88-NF-*κ*B signaling pathway and exacerbates mitochondrial dysfunction.

## 1. Introduction

Gout is one of the most common forms of inflammatory arthritis in adults and is induced by MSU crystals deposited within the joints [1]. In the long-term hyperuricemia, MSU crystals deposit, usually with tophi, further trigger a chronic inflammatory response that may result in structural damage of the joint, often referred to as gout arthritis (GA) or chronic gout [2]. Nonsteroidal anti-inflammatory drugs, colchicine, and glucocorticoids are commonly used to relieve acute pain and inflammation. In addition, the long-term use of these drugs is limited by the inevitable side effects of gastrointestinal toxicity and nephrotoxicity, as well as therapeutic gaps [3, 4]. However, further study of the biology of inflammation may open the way for new treatment strategies of gout.

The inflammatory response to gout involves cell-surface receptor-mediated signaling pathways that coordinate the innate immune response. The most important of these receptors is TLR4, which is particularly involved in gout inflammation [5]. Although heat shock proteins are generally considered to be intracellular proteins, it has been reported that heat shock proteins are released from cultured cells [6], and in particular, both Hsp60 and Hsp72 have been detected in normal human plasma [7, 8]. Serum HSP60 level is elevated in patients with inflammatory diseases such as diabetes [9], colitis [10], and acute lung injury [11]. It has been reported that the HSP60 antibody inhibits arthritis and colitis in mice and balances cytokines toward anti-inflammatory response [12]. In addition, Hsp60 has been shown to activate macrophages through TLR4, inducing an inflammation cascade [13].

Because NLRP3 inflammasome is a key component in the response to MSU crystals, strategies that prevent their activation or affect their activity can relieve gout inflammation. It has been demonstrated that mitochondrial dysfunction is closely related to the activation of NLRP3 inflammasome [14]. Hsp60 is a molecular stress protein mainly located in mitochondria, which plays the function of protein folding in the mitochondrial matrix. Hsp60 is upregulated in the presence of mitochondrial damage and is considered an indicator of mitochondrial stress [15]. A previous study indicated that HSP60 overexpression promoted mitochondrial dysfunction and activated the NLRP3 inflammasome pathway in JEV infection [16]. As we observed the important role of HSP60 in the TLR4 signaling pathway and NLRP3 inflammasome activity, this prompted us to investigate its role in gout. However, whether HSP60 is involved in MSU crystal-induced inflammation and its molecular mechanism remains unclear. In the current study, we explored the role of HSP60 in MSU crystal-induced inflammation and its possible molecular mechanism.

## 2. Materials and Methods

### 2.1. Patients

All participants were gout patients from the Department of Rheumatology of The Affiliated Hospital of North Sichuan Medical College. Patients with acute gout (AG) must fall within the classification criteria of the American College of Rheumatology (ACR). Intercritical gout (IG) is defined as complete remission of acute gout and normal C-reactive protein (CRP) or erythrocyte sedimentation rate. In addition, patients had no history of infection, hematopathy, cancer, nephropathy, or other autoimmune diseases. Age-matched males served as healthy controls (HC) for regular physical examinations at the Affiliated Hospital of North Sichuan Medical College during the same period. Patient characteristics are shown in Table 1. This study was approved by the Ethics Committee of the Affiliated Hospital of North Sichuan Medical College, and all patients were required to provide informed consent in order to participate in the study.

### 2.2. PBMC Harvest

After PBMCs were isolated from blood samples of gout patients or HCs through density gradient centrifugation and stored at -80°C for gene- and protein-related tests.

### 2.3. Plasmid Construction

Human (HSPD1, NM_199440.2) and Mice HSP60 (HSPD1, NM_010477.4) cDNA open reading frame were, respectively, cloned into pcDNA3.1 vector and sequenced to exclude mutations; the vector also contained a green fluorescence protein- (GFP-) coding sequence.

### 2.4. Preparation of MSU Suspension

After, RAW264.7 cells and THP-1-derived macrophages were transfected with siRNA or plasmid and then treated with MSU crystals and determined by cytokines. MSU crystals were prepared as previously described [17]. 1 g uric acid (Sigma-Aldrich, St. Louis, MO, USA) was dissolved in 200 mL boiling water containing 1 n NaOH, adjusted to pH 8.9 by adding NaOH, and crystallized overnight at room temperature. The precipitation was filtered out of the solution and dried at 42°C. The crystals were weighed under sterile conditions and suspended in a concentration of 25 mg/mL PBS. RAW264.7 cells were cultured with DMEM containing 10% FBS. THP-1 cells were seeded in 6-well culture plates with RPMI 1640 culture medium containing 10% fetal bovine serum (FBS) and administrated with phorbol myristate acetate (PMA, 100 ng/ml) for 24 h to obtain THP-1-derived macrophages. After THP-1-derived macrophages or RAW264.7 cells were transfected with pcDNA3.1-HSP60 plasmid or pcDNA3.1 vector for 36 h, the cells were primed with LPS (100 ng/ml) for 1 hr and subsequently challenged with MSU suspension. After mouse or human HSP60 gene siRNA was transfected into RAW264.7 cells or THP-1-derived macrophages for 48 h, primed with LPS, and then treated with MSU suspension, siRNA sequences were synthesized by Sango Biotech (Shanghai, China). Mouse HSP60 siRNA sequence: 5′-GGCUGUGGAUGCUGUAAUUTT-3′ (sense), 5′-AAUUACAGCAUCCACAGCCTT-3′ (antisense). Human HSP60 siRNA sequence: 5′-AGUCAAGGCUCCAGGGUUUTT-3′ (sense), 5′-AAACCCUGGAGCCUUGACUTT-3′ (antisense). Transfection was performed by using Lipofectamine® LTX Reagent (Thermo Fisher Scientific) according to the manufacturer's instructions. The supernatants were applied to measure IL-1*β*, TNF-*α*, IL-6, and PGE2 levels using ELISA (Neobioscience kit, Shenzhen, China) following the manufacturer's instruction.

### 2.5. Western Blot Analysis

RIPA buffer was applied to extract total protein from either macrophages or foot pad tissues. Cytoplasmic and Mitochondrial Proteins were extracted using Cytoplasmic and Mitochondrial Protein Extraction Kit (Sango Biotech, Shanghai, China) following the manufacturer's instruction. Cell culture supernatants were collected and concentrated (×10) using Amicon Ultra Centrifugal Filters (MilliporeSigma). Proteins were suspended in Laemmli Buffer 1×, boiled at 95°C for 6 minutes, and resolved by SDS-PAGE. Then, proteins were transferred to PVDF membrane. Western blot was performed using the following antibodies: anti-IL-1*β*, anti-Phospho-NF-*κ*B p65(Ser536), and anti-Phospho-NF-*κ*B p105/50 (Ser933) were purchased from Cell Signaling Technology (CST, USA), and anti-COX-2, anti-iNOS, anti-MPO, anti-I*κ*B*α*,, anti-MyD88, anti-Caspase-1, anti-TLR4, anti-NF-*κ*B p65, anti-NF-*κ*B p105/50, and anti-NLRP3 were obtained from HuaBio (Hangzhou, China). The membrane was incubated with the primary antibodies at 4°C overnight. After washing the membrane, the membrane was incubated with second antibodies (1 : 7500 dilution, Boster, Wuhan, China) for 1 h at room temperature. Finally, the membrane was exposed to the gel imaging system with a chemiluminescence kit. The band intensity was quantified using Image J software.

### 2.6. TLR4 Inhibitor Treatment

After RAW264.7 cells were transfected with pcDNA3.1-HSP60 plasmid or pcDNA3.1 vector for 36 h, cells were treated with 0 or 5 *μ*M CLI-095 (TLR4 inhibit, Invitrogen) for 2 h and then primed with LPS for 1 h prior to MSU crystal stimulation.

### 2.7. Mitochondria-Targeted Antioxidant Treatment

After THP-1-derived macrophages were transfected with pcDNA3.1-HSP60 plasmid or pcDNA3.1 vector for 36 h, cells were added 0 or 50 nM MitoTEMPO (Cayman Chemical) for 4 h and then primed with LPS for 1 h prior to MSU crystal stimulation.

### 2.8. RNA Extraction and Quantitative Real-Time PCR

Total RNA was extracted from macrophages using TRIzol reagent (Invitrogen) and reverse-transcribed into cDNA using reverse transcription reagents (TaKaRa). Real-time quantitative PCR (RT-qPCR) was performed using the SYBR Green I Two-Step qRT-PCR kit with ROX (Invitrogen), and *β*-actin as an internal reference. Target gene expression was analyzed through the 2^−△△CT^ method. Primer sequences are shown in Table 2.

### 2.9. Measurement of Cellular Antioxidant Enzyme Activity

After THP-1-derived macrophages were transfected with human HSP60 specific siRNA for 48 h, primed with LPS, and then treated with MSU crystals for 12 h, THP-1-derived macrophages cells were washed with cold PBS for three times, then added lysis buffer (20 mM Tris buffer pH 7.5, 150 mM NaCl, and 1% Triton X-100) at 4°C for 15 min. The cell lysates were collected after centrifugation at 12000 g and 4°C for 15 min. The intracellular activities of SOD, GSH-Px, and CAT of the cell lysates were tested with the corresponding assay kits (Beyotime, China) following the manufacturer's instructions. The protein content was measured by a BCA assay kit (Thermo Fisher Scientific) according to the manufacturer's protocol, and the results were expressed as U/mg protein.

### 2.10. MSU Crystals Induced Mouse Model of Arthritis Treatment with Vivo Morpholino

All experimental procedures were approved by North Sichuan Medical College. HSP60 vivo-morpholino (HSP60-Mo) and control morpholino (Ctrl Mo) oligos were purchased from Gene Tools LLC (Philomath, OR, USA). HSP60-Mo was designed against sequences of mouse HSP60 (HSPD1) gene to specifically target it (5′-AGGACTGTGGGTAGTCGAAGCATTT-3′). A 25-base scrambled morpholino of random sequence (Ctrl Mo) was used as a negative control (5′-CCTCTTACCTCAGTTACAATTTATA-3′). Vivo-morpholino (15 mg/kg body weight) was injected into the right pad, and PBS (the same volume) was injected into the left pad of the same mouse. After 1 h, 1 mg of MSU (in 40 *μ*l of PBS) was injected into the right foot pad, and an equal PBS volume was simultaneously injected into the left foot pad as the control. Joint index evaluation is based on a previously described method [15]. Paw joint swelling was measured 24 h after MSU suspension injection. The swelling index was expressed as the ratio of injected MSU+vivo-morpholino or MSU joint to injected PBS joint in the same mouse. The mice were sacrificed afterward. Foot pad tissues were homogenized in RIPA buffer, and the supernatants were used to measure the related protein levels and myeloperoxidase (MPO) activity, in order to quantify neutrophil sequestration. This was done using an MPO activity assay kit (Jiancheng Technology Co, Nanjing, China) and foot pad tissue homogenates, according to the manufacturer's guidelines. Foot pad tissue sections were prepared for hematoxylin and eosin (H&E) staining and immunostaining.

### 2.11. Mitochondrial ROS and Membrane Potential Staining Detection

Mitochondrial superoxide indicator (Mito-SOX red, 40778ES50) and Mito-Tracker Green (40742ES50) were purchased from YEASEN (Shanghai, China). Cells were stained with the Mito-SOX red (5 *μ*M) and Mito-Tracker Green (100 nM) for 30 min at 37°C/5% CO2 in the dark and subsequently washed with PBS to remove free probes. Then, nuclei were stained with Hoechst 33342 (5 *μ*g/ml, Yeasen, Shanghai, China, 40731ES10) for 10 min at 37°C/5% CO2 in the dark and washed with PBS. ROS quantification was represented by the ratio of Mito-SOX red to Mito-Tracker Green. Mitochondrial membrane potential (MMP) was evaluated by the MMP assay kit with JC-1 (Beyotime, Shanghai, China, C2006) following the manufacturer's guidelines protocol. Cells were washed with PBS and were then stained with JC-1 probe for 20 min at 37°C/5% CO2 in the dark. Subsequently, dye buffer was used to remove free probes. The red-to-green fluorescence ratio was used to analyze the changes of MMP. The cells were live-imaged immediately after incubation with fresh complete culture medium for 60 min using the Olympus Laser Scanning Confocal Microscope. The excitation and emission wavelengths for each fluorescent dye were selected according to the manufacturer's instructions. The fluorescence intensity was analyzed by Image J software.

### 2.12. Mitochondrial DNA Measurement

Relative mitochondrial DNA level was detected by quantitative PCR using SYBR Green PCR Master Mix (Invitrogen). The quantification of mtDNA was performed as described previously [18]. Two independent reactions were operated using mitochondrial and nuclear gene primers. MtDNA copy number was detected by quantitative PCR and normalized to the nuclear DNA level according to the ratio of cytochrome C oxidase 1 (mtCOI) DNA to nuclear DNA (encoding 18S ribosomal RNA). The primers sequence was provided in Table 2.

For the determination of mtDNA in cytoplasm, 1 × 10^7^ cells were homogenized with 100 mM Tricine-NaOH solution, pH 7.4 containing 0.25 M sucrose, 1 mM EDTA, and protease inhibitor and centrifuged at 700 g at 4°C for 10 min. The protein concentration and volume of the supernatant were normalized and centrifuged at 10,000 g for 30 min at 4°C to obtain the supernatant corresponding to the cytoplasmic components. As described above, DNA was isolated from 200 *μ*L cytoplasmic components using a Tiangen DNA extraction kit.

### 2.13. Mitochondrial ATP Detection Assay

Mitochondrial ATP levels were detected using the ATP assay kit (Beyotime, China) according to the manufacturer's protocol. The contents of ATP were analyzed from 3 independent experiments and detected by a multiscan spectrum (SynergyH1MD, BioTek). The results were expressed in terms of chemiluminescence intensity compared with control.

### 2.14. Cell and Tissue Section Immunofluorescence

For mitochondria and HSP60 colocalization, the cells were first stained with Mito-Tracker Green and fixed at room temperature with 4% paraformaldehyde for 10 min, followed by incubation with rabbit anti-mouse or anti-human HSP60 antibody (1 : 150 HUABIO, Hangzhou, China) diluted in 1% BSA/PBS (PBS, pH 7.6) overnight at 4°C. A secondary antibody (Alexa Fluor 488-conjugated Affinipure Goat) and DAPI staining solution were both purchased from Beyotime (Shanghai, China). Mouse foot pad tissue sections were analyzed by immunofluorescence. Rabbit anti-HSP60 antibody (HUABIO), rabbit anti-Ly6G antibody (HUABIO), and rabbit anti-MPO antibody (Boster, Wuhan, China) were used. Images were acquired using an Olympus Laser Confocal Microscope and analyzed by Image J software.

### 2.15. Statistics

Statistical analysis was performed using GraphPad Prism 6 software. Data were expressed as the mean ± SEM. One-way ANOVA analysis of variance was used for statistical analysis. *p* < 0.05 was regarded as significantly different by using LSD or Dunnett's T3 test.

## 3. Results

### 3.1. HSP60 Expression Was Upregulated in Gout Patients

**Figure 1:**
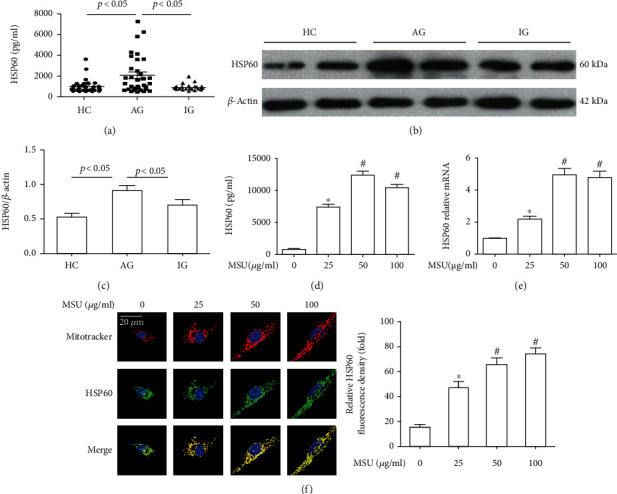
HSP60 expression was upregulated in gout and MSU crystal-stimulated THP-1-derived macrophages. (a) HSP60 protein level in the plasma from patients with AGA (*n* = 31), IGA (*n* = 33), and HC (*n* = 43) was detected by ELISA. (b) HSP60 protein level in PBMCs of patients with AGA, IGA, and HCs was measured by western blot (8 cases in each group). (c) Densitometry analysis of HSP60 protein level in PBMCs. (d–f) THP-1-derived macrophages were primed with LPS (100 ng/ml) for 1 h and then treated with different concentrations of MSU suspension for 12 h. (d) HSP60 secretion was analyzed by ELISA in THP-1-derived macrophages. (e) The relative mRNA expression of HSP60 in MSU crystal-stimulated THP-1-derived macrophages. (f) Representative images of double-labeling HSP60 and mitochondrial marker (MitoTracker) in MSU crystal-stimulated THP-1-derived macrophages. Scale bar: 20 *μ*m. Blue shows nuclei staining with DAPI, quantification of immunofluorescence staining of HSP60 using Image J software. ^∗^In comparison with absence of MSU crystal treatment and ^#^ in comparison with 25 *μ*g/ml MSU crystal treatment.

In this study, to confirm HSP60 expression in patients with gout, HSP60 expression levels in serum and PBMCs were detected. Serum HSP60 levels were significantly higher in patients with AG than that in IG or HC (Figure 1(a)). In PBMCs, HSP60 protein levels in gout patients were higher compared to HCs (Figures 1(b) and 1(c)).

### 3.2. MSU Crystals Accelerated HSP60 Expression in THP-1-Derived Macrophages and RAW264.7 Cells

To explore the effect of MSU crystals on HSP60 secretion in THP-1-derived macrophages, cells were exposed to varying doses of MSU crystals for 12 h. Extracellular HSP60 levels were detected by ELISA in the culture supernatants, revealing an increased level of HSP60 secretion upon MSU crystals treatment (Figure 1(d)). As shown in Figure 1(e), RT-PCR data also displayed that MSU crystals promoted the expression of HSP60 at mRNA level almost in a dose-dependent manner. To further investigate the effect of MSU crystal on the localization of HSP60 in the macrophage, double staining of MitoTracker and immunofluorescence was used to analyze HSP60 distribution in the mitochondria and cytoplasm. The data revealed that HSP60 was mainly distributed in the mitochondria of macrophages, and a small amount was distributed in the cytoplasm in THP-1-derived macrophages. When MSU crystals were used to treat THP-1-derived macrophages, in low-concentration MSU crystals, almost all HSP60 proteins were located in the mitochondria (Figure 1(f)).

We further assessed the effect of different concentrations of MSU crystals on the distribution and secretion of HSP60 in the RAW264.7 cells. The results revealed that MSU crystals also had a great influence on the distribution and secretion of HSP60, and HSP60 protein levels reached a peak in RAW264.7 cells exposed to 100 *μ*g/ml of MSU crystals (Supplementary Figures 1(a) and 1(b)). HSP60 protein levels in the cytoplasm or mitochondria were further assessed using western blot from RAW264.7 cells stimulated by different concentrations of MSU crystals (Supplementary Figure 1(c)). MSU crystals almost dose-dependently upregulated the expression of HSP60 in the mitochondria, and the protein levels of HSP60 almost reach to peak in RAW264.7 cells treatment with 100 *μ*g/ml of MSU crystals. In RAW264.7 cells exposed to MSU crystals, HSP60 mRNA expression and secretion increased significantly almost in a time-dependent manner (Supplementary Figures 2(a) and 2(b)).

### 3.3. HSP60 Regulates TLR4/MyD88/NF-*κ*B Signaling Pathway in MSU Crystal-Stimulated Inflammation

**Figure 2:**
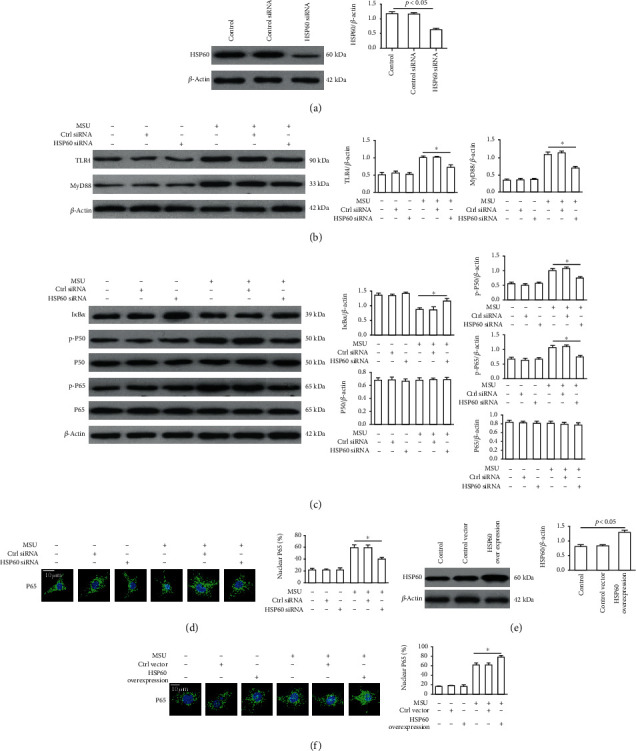
HSP60 affects the TLR4/MyD88/NF-*κ*B signaling pathway in MSU crystal-stimulated RAW264.7 cells. After RAW264.7 cells were transfected with HSP60 siRNA or control siRNA for 48 h, primed with LPS (100 ng/ml) for 1 h, and then treated with MSU crystals (100 *μ*g/ml) for 4 h. (a) HSP60 protein level in RAW264.7 cells transfected with ctrl siRNA or HSP60 siRNA. (b, c) Protein levels of TLR4, MyD88, I*κ*B*α*, P50, P65, phosphorylated P50 (p-P50), and phosphorylated P65 (p-P65). (d) HSP60 knockdown inhibited MSU crystal-induced P65 nuclear localization in RAW264.7 cells and analyzed by immunostaining. Blue shows nuclei staining with DAPI. Scale bar: 10 *μ*m. Each experiment had six fields of view. The intensity of the fluorescence signal was analyzed by Image J software. (e) HSP60 protein level in RAW264.7 cell transfection with control vector or HSP60 vector for 36 h. (f) RAW264.7 cells were transfected with a control vector or HSP60 vector for 36 h, primed with LPS (100 ng/ml) for 1 h, primed with LPS (100 ng/m), and then treated with MSU crystals (100 *μ*g/ml) for 4 h. HSP60 overexpression promoted MSU crystal-induced P65 nuclear localization in RAW264.7 cells and analyzed by immunostaining. Blue shows nuclei staining with DAPI. Scale bar: 10 *μ*m. Each experiment had six fields of view. The intensity of the fluorescence signal was analyzed by Image J software. Values are the mean ± SEM of 3 independent experiments. ^∗^*p* < 0.05.

In our study, we set out to explore the effect of HSP60 on the TLR4/MyD88/NF-*κ*B signaling pathway. Specific siRNA was used to interfere with HSP60 expression in RAW264.7 cells for 48 h (Figure 2(a)), followed by a 4 h MSU crystals treatment. Compared with control siRNA, the expression of TLR4 and its intracellular junction protein (MyD88) in RAW264.7 cells treatment with MSU crystals were inhibited due to the downregulation of HSP60 (Figure 2(b)). The MyD88 signaling pathway is closely related to I*κ*B*α* degradation and the phosphorylation of NF-*κ*B in the MSU crystal-induced inflammation. As a result of HSP60 knockdown, the I*κ*B*α* degradation and the phosphorylation of NF-*κ*B P65 and P50 showed a reduction in MSU crystals exposed to RAW264.7 cells (Figure 2(c)).

To explore the involvement of HSP60 in the regulation of the TLR4 signaling pathway in MSU crystal-induced inflammatory response, we inhibited TLR4 signaling using a specific TLR4 signaling inhibitor (CLI-095, InvivoGen) in RAW264.7 cells as described in methods. The levels of phosphorylation of P65 (p-P65) and P50 (p-P50) were checked by western blot. As shown in Supplementary Figure 3, TLR4 inhibitor (CLI-095) reversed the enhanced p-P65 and p-P50 protein levels due to HSP60 overexpression in MSU crystal-induced RAW264.7 cells.

Phosphorylation of NF-*κ*B induces its nuclear localization, which is important for regulating inflammatory gene expression. Therefore, we sought to evaluate the impact of both HSP60 knockdown and HSP60 overexpression on the nuclear localization of phosphorylated NF-*κ*B P65 using immunofluorescence in RAW264.7 cells. Nuclear translocation of phosphorylated NF-*κ*B P65 triggered by MSU crystals was attenuated by HSP60 knockdown (Figure 2(d)). RAW264.7 cells were transfected with HSP60 vector or empty vector for 48 h and then treated with MSU crystals for 4 h. Western blot analysis was used to demonstrate HSP60 overexpression (Figure 2(e)). HSP60 overexpression further accelerated the nuclear localization of phosphorylated NF-*κ*B P65 induced by MSU crystals (Figure 2(f)). These results imply that HSP60 plays a significant role in the MSU crystal-stimulated nuclear localization of phosphorylated NF-*κ*B subunit.

### 3.4. HSP60 Influences the Expression of NF-*κ*B Downstream Inflammatory Associated Gene Induced by MSU Crystals

**Figure 3:**
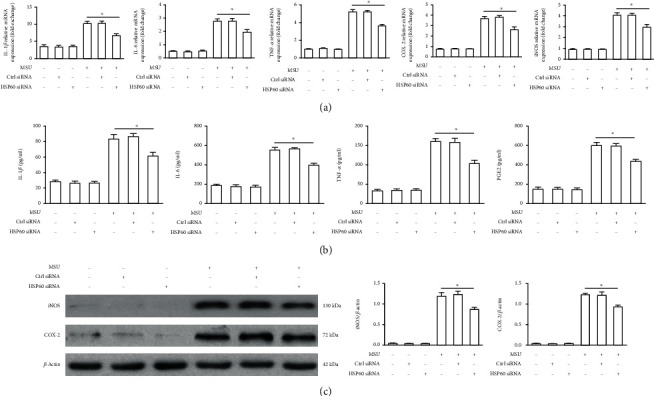
HSP60 knockdown reduced the expression of MSU crystal-induced inflammatory associated gene in THP-1-derived macrophages. THP-1-derived macrophages were transfected with ctrl siRNA or HSP60 siRNA for 48 h, primed with LPS (100 ng/ml) for 1 h, and then stimulated with MSU crystals (50 *μ*g/ml) for 12 h. (a) Quantification of RT-PCR analysis of IL-1*β*, IL-6, TNF*α*, COX-2, and iNOS mRNA expression. (b) The release of IL-1*β*, IL-6, TNF*α*, and PGE2 in the culture supernatant was measured by ELISA. (c) Western blot analysis of COX-2 and iNOS protein levels. Data are represented as the mean ± SEM for three experiments. ^∗^*p* < 0.05.

NF-*κ*B activation impels the transcription of cytokines and prompts a complex inflammatory cascade response. As shown in Figure 3(a), reduction in endogenous HSP60 expression by specific siRNA in THP-1-derived macrophages blocked mRNA expression levels of MSU crystal-induced proinflammatory enzymes (COX-2 and iNOS) and proinflammatory cytokines (IL-1*β*, IL-6, and TNF-*α*) (Figure 3(a)). In accordance with the RT-PCR results, ELISA data indicated that lower IL-1*β*, TNF-*α*, IL-6, and PGE2 protein levels were found in the cell culture supernatants of HSP60 knockdown THP-1-derived macrophages in response to MSU crystals (Figure 3(b)), and western blot analysis data also revealed that MSU crystal-induced iNOS and COX-2 protein levels were also suppressed in HSP60 knockdown THP-1-derived macrophages (Figure 3(c)). All these data suggest that HSP60 has a positive effect on the production of MSU crystal-induced proinflammatory cytokines.

### 3.5. HSP60 Regulates NLRP3 Inflammasome Activation Triggered by MSU Crystals

**Figure 4:**
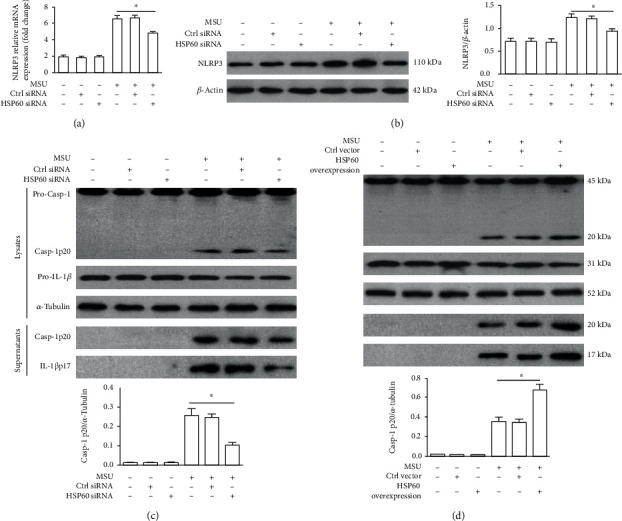
HSP60 affects the activation of the NLRP3 inflammasome in MSU crystal-treated THP-1-derived macrophages. (a–c) THP-1-derived macrophages were transfected with control siRNA or HSP60 siRNA for 48 h, primed with LPS (100 ng/ml) for 1 h, and then treated with MSU suspension (50 *μ*g/ml) for 12 h. (a) Effect of HSP60 knockdown on NLRP3 mRNA levels. (b) Influence effect of HSP60 knockdown on NLRP3 protein levels. (c) Effect of HSP60 knockdown on the expression of the p20 subunit of Caspase-1 and IL-1*β* in the cell lysates and culture supernatants. (d) THP-1-derived macrophages were transfected with a control vector or human HSP60 vector for 36 h, primed with LPS (100 ng/ml) for 1 h, and then treated with MSU suspension (50 *μ*g/ml) for 12 h. Effect of HSP60 overexpression on the expression of the p20 subunit of Caspase-1 and IL-1*β* in the cell lysates and culture supernatants. The p20 subunit of Caspase-1 protein levels in the cell lysates was normalized to ɑ-Tubulin and then averaged. Values are the mean ± SEM of 3 independent experiments. ^∗^*p* < 0.05.

Previous studies have indicated that NLRP3 inflammasome activation is an important pathway by which MSU crystals lead to cellular inflammatory response, and nuclear localization of phosphorylated NF-*κ*B promotes NLRP3 inflammasome activation by inducing transcription of the NLRP3 gene. Therefore, we investigated the role of HSP60 in NLRP3 expression induced by MSU crystals using RT-PCR and western blot. MSU crystal-induced NLRP3 expression both at mRNA and protein levels was decreased because of HSP60 knockdown (Figures 4(a) and 4(b)). NLRP3 inflammasome activation contributes to the cleavage of pro-Caspase-1 to Caspase-1 and the release of IL-1*β* [19]. Caspase-1 activation and IL-1*β* release were alleviated because of HSP60 knockdown in the MSU crystals stimulated THP-1-derived macrophages (Figure 4(c)). Oppositely, pro-Caspase-1 cleavage into Caspase-1 and IL-1*β* production in cell lysates and culture supernatants observed an obvious increase in THP-1-derived macrophages stimulated by MSU crystals due to HSP60 overexpression (Figure 4(d)).

### 3.6. HSP60 Aggravates Mitochondrial Dysfunction Induced by MSU Crystals

**Figure 5:**
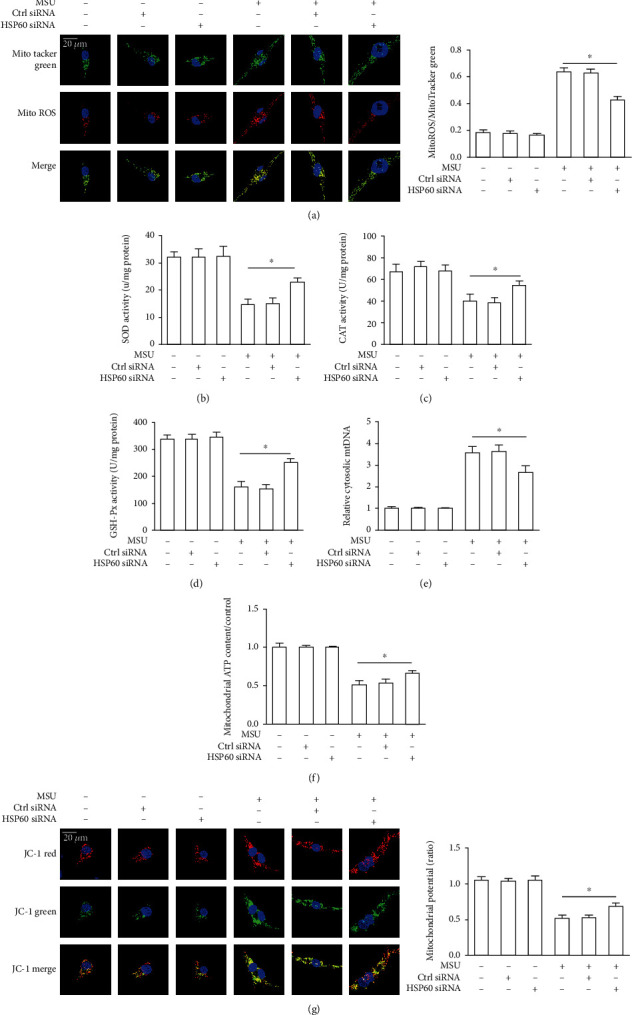
HSP60 knockdown alleviates mitochondrial dysfunction in MSU crystal-stimulated THP-1-derived macrophages. (a–g) THP-1-derived macrophages were transfected with control siRNA or HSP60 siRNA for 48 h, primed with LPS (100 ng/ml) for 1 h, and then treated with MSU suspension (50 *μ*g/ml) for 12 h. (a) The effect of HSP60 knockdown on the mitochondrial ROS, representative images of MitoTracker green and MitoROS staining. Blue shows nuclei staining with Hoechst33342. Scale bar: 20 *μ*m. (b) SOD activity. (c) CAT activity. (d) GSH-Px activity. (e) Mitochondrial DNA release was detected by quantitative real-time PCR analysis. (f) Relative mitochondrial ATP content. (g) The effect of HSP60 knockdown on the mitochondrial membrane potential (MMP), cells were stained using JC-1 probe. Blue shows nuclei staining with Hoechst33342. Scale bar: 20 *μ*m. For mtROS and MMP analysis, >50 individual cells were imaged per group from 3 culture dishes. Data are representative of mean ± SEM for three experiments. Each experiment had 10 fields of view. ^∗^*p* < 0.05.

HSP60 expression increases when mitochondria are damaged, and HSP60 is considered an indicator of mitochondrial stress [13]. Mitochondrial dysfunction, which includes mitochondrial membrane potential collapse, overproduction of mitochondrial reactive oxygen species (mtROS), and release of mitochondrial DNA, is crucial for the activation of the NLRP3 inflammasome. Therefore, we sought to explore the role of HSP60 in the mitochondrial damage induced by MSU crystals. MitoSOX Red is a mitochondrial superoxide indicator that is inserted into mitochondrial DNA during oxidation and produces red fluorescence. MitoTracker Green was used to determine the total mitochondrial content. The red to green fluorescence ratio reflects the mtROS level. HSP60 knockdown resulted in decreased mtROS level in MSU crystal-stimulated THP-1-derived macrophages and RAW264.7 cells, supporting lower levels of superoxide production (Figure 5(a), Supplementary Figure 4(a)). However, mtROS generation was aggravated due to HSP60 overexpression (Supplementary Figure 4(b)). In order to investigate the involvement of HSP60 in oxidative stress induced by MSU crystals, we further explored the effect of inhibition of HSP60 expression on the activity of intracellular antioxidant enzymes in THP-1-derived macrophages. The activities of CAT, GSH-Px, and SOD were significantly increased because of HSP60 knockdown in MSU crystals stimulated THP-1-derived macrophages (Figures 5(b)–5(d)), which further hinted that HSP60 had the function of regulating the production of mtROS induced by MSU crystals. We also found a reduction in mitochondrial DNA release to cytosol in HSP60 knockdown RAW264.7 cells in response to MSU crystals (Figure 5(e)). Next, we measured the mitochondrial ATP levels in MSU crystal-induced THP-1-derived macrophages and found a dramatic decrease in mitochondrial ATP levels because of MSU stimulation. However, a great increase in mitochondrial ATP levels was observed because of HSP60 knockdown in MSU crystal-induced THP-1-derived macrophages (Figure 5(f)).

The mitochondria will be damaged by increased reactive oxygen species in the mitochondria. To evaluate the level of mitochondrial damage, the mitochondrial membrane potential (*ΔΨ*m) was detected using the fluorescent probe, JC-1. When the mitochondrial membrane potential is high, JC-1 gathered in the mitochondrial matrix and formed a red fluorescent polymer (J-aggregates), whereas the mitochondrial membrane potential is low, JC-1 cannot be concentrated in the mitochondrial matrix. In this case, JC-1 is a monomer and can produce green fluorescence. The ratio of red to green fluorescence is used to calculate mitochondrial membrane potential and reflects the levels of polarized functional mitochondria. Because of HSP60 knockdown, mitochondrial membrane potential was restored in MSU crystal-induced THP-1-derived macrophages and RAW264.7 cells, supporting enhanced mitochondrial function (Figure 5(g), Supplementary Figure 4(c)). On the other hand, we observed a further reduction in MMP collapse triggered by MSU crystal-stimulated RAW264.7 cells due to HSP60 overexpression (Supplementary Figure 4(d)).

To investigate the relationship between HSP60 induced mitochondrial dysfunction and NLRP3 inflammasome activation in MSU crystal-induced inflammation, we used mitochondria-targeted antioxidant (MitoTEMPO) to improve mitochondrial function in THP-1-derived macrophages as described in methods. The release of IL-1*β* in the culture supernatants was detected by western blot and ELISA. As shown in Supplementary Figure 5(a), MitoTEMPO reversed the increase of IL-1*β* release because of HSP60 overexpression in MSU crystal-induced THP-1-derived macrophages. In line with western blot data, the ELISA result displayed that as a result of the addition of MitoTEMPO, we observed an obvious reduction of IL-1*β* level (Supplementary Figure 5(b)). These data suggested that HSP60 might promote the activation of NLRP3 inflammasome by affecting mitochondrial function.

### 3.7. HSP60 Downregulation Alleviates the Severity of MSU Crystals Induced Mouse Model of Arthritis

**Figure 6:**
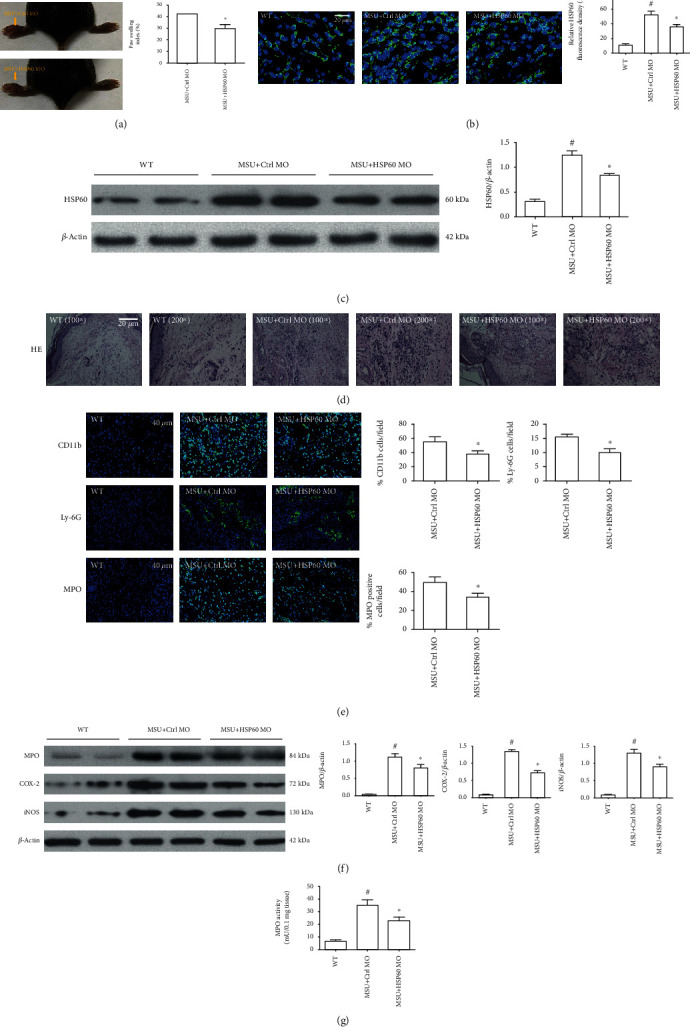
HSP60 downregulation relieved the severity of MSU crystal-induced inflammatory mouse model. (a) Mice injected with HSP60-MO presented decreased the paw swelling index (*n* = 6 per group, mean ± SEM). (b) Immunostaining was used to detect HSP60 expression in the foot pad tissue section (*n* = 4 per group, mean ± SEM). Blue shows nuclei staining with DAPI. Scale bar: 20 *μ*m. (c) HSP60 protein levels were analyzed by western blot in the foot pad tissue (*n* = 4 per group, mean ± SEM). (d) HE staining was used to observe the infiltration of inflammatory cells in the foot pad tissue sections (*n* = 4 per group, mean ± SEM). Images were obtained using a light microscope (Olympus, Tokyo, Japan) with 10 times or 20 times objectives and analyzed with image processing software (Olympus). (e) Immunostaining using anti-CD11b, anti-Ly-6G, and anti-MPO antibody in the foot pad tissue sections (*n* = 4 per group, mean ± SEM). Blue shows nuclei staining with DAPI. Scale bar: 40 *μ*m. (f) Protein levels of MPO, iNOS, and COX-2 in the foot pad tissue were measured by western blot (*n* = 4 per group, mean ± SEM). (g) The MPO activity of foot pad tissue (*n* = 6 per group, mean ± SEM). ^#^Significantly different from the absence of MSU crystals injection mice and ^∗^ significantly different from MSU crystals and ctrl MO injection mice.

We proceeded to study the role of HSP60 in the mouse model of gouty arthritis triggered by MSU crystals. After mice were, respectively, injected with HSP60-MO or ctrl-MO into the right foot pad tissue for 1 h, MSU suspensions were, respectively, injected into the right foot pad of mice to simulate the etiology of human gouty arthritis. A significant decrease in paw swelling index was noted in MSU+HSP60-MO-treated mice compared to MSU+ctrl-MO-treated mice (Figure 6(a)). Both immunostaining and western blot data indicated that HSP60 expression levels were significantly lower in MSU+HSP60-MO-treated mice compared to MSU+ctrl-MO-treated mice (Figures 6(b) and 6(c)). Histological analyses of the foot pad tissue section revealed that HSP60 knockdown prevented the infiltration of inflammatory cells into the foot pad tissue (Figure 6(d)). We further confirmed the effect of downregulated HSP60 expression on neutrophils, myeloperoxidases, and macrophage distribution in the foot pad tissue section through immunostaining. As depicted in Figure 6(e), a decreased number of neutrophils and macrophages in the footpad tissue section were observed in the MSU+HSP6-MO treated mice. Concomitantly, the amount of MPO containing cells was also significantly lower in the MSU+HSP60-MO-treated mice compared to MSU+ctrl-MO-treated mice (Figure 6(e)). We further assessed the protein levels of important proinflammatory enzymes (MPO, iNOS, and COX2) in the foot pad tissue extracts using western blot. The MPO, iNOS, and COX-2 protein levels from the foot pad tissue extracts in HSP60-MO treated mice were found to be greatly lower (Figure 6(f)). We also observed a decrease of NLRP3, TLR4, and MyD88 protein levels from the foot pad tissue extracts in HSP60-MO-treated mice (Supplementary Figures 6(a)–6(d)). Compare to MSU+crtl-MO mice, the activity of MPO also revealed to be decreased in the MSU+HSP60-MO-treated mice, reflecting lower levels of inflammation. HSP60-deficient mice had less ankle joint swelling compared to the ctrl vivo-morpholino-treated group, which was consistent with the reduction in observed swelling of the foot pad (Figure 6(g)). These results suggested that HSP60 knockdown relieved MSU crystal-induced inflammation in vivo.

## 4. Discussion

Previous studies imply that HSP60 has the potential to serve as a biomarker and therapeutic target for diagnosis and patient monitoring in many inflammatory conditions [20, 21]. In certain pathological conditions, such as bronchitis [22], keratoconjunctivitis [23], hepatitis [24], thyroiditis [25], periodontitis [26], and IBD [10], the upregulation of Hsp60 is a part of excessive inflammatory response. Hsp60 can survive and function in a variety of intracellular and extracellular environments, and its canonical location is the mitochondrial matrix. Although the exact mechanism by which HSP60 is secreted to the extracellular medium remains unknown, the existence and function of Hsp60 in extramitochondrial compartments have been stated [27]. In the present study, our data indicates that HSP60 serum level in gout patients is higher than that in healthy individuals. Both in human- and mouse-derived macrophages, MSU crystals can significantly accelerate HSP60 secretion. HSP60 was low expressed in the cytoplasm of THP-1-derived macrophages and RAW264.7 cells. However, after MSU crystal stimulation, the HSP60 level in the cytoplasm was further reduced, but it was significantly increased in mitochondria. The molecular mechanism by which MSU crystals promote the secretion of HSP60 and increase the expression level in mitochondria remains to be further explored. These data suggest that HSP60 may be released extracellular or aggregated in mitochondria to modulate inflammation induced by MSU crystals.

Previous reports have shown that extracellular HSP60 can activate macrophages through TLR4 [28]. When TLR4 is activated, it can coordinate multiple signaling cascades, including arthritis animal models and in patients with gout [29–31]. However, the role of HSP60 in the TLR4-related signaling pathway induced by MSU crystals is elusive. In our study, significant increases in the expression of HSP60, TLR4, and its adaptor protein MyD88 were observed after MSU crystal stimulation, suggesting that MSU crystals promoted enhanced HSP60 expression in macrophages and then upregulated TLR4 and MyD88 expression. Activated TLR4 recruits MyD88 by interacting with the TLR domain, initiating an intracellular signaling cascade that leads to the activation of the downstream transcription factor NF-*κ*B and the production of proinflammatory factors. HSP60 knockdown or overexpression can affect the phosphorylation and nuclear translocation of NF-*κ*B subunits, as well as the expression of its downstream inflammatory genes in MSU crystals induced inflammation. Phosphorylation levels of P65 and P50 were significantly reduced after TLR4 signaling was suppressed. All these imply that HSP60 may regulate the activation of NF-*κ*B through the TLR4 signaling pathway in MSU crystal-induced inflammation. But the exact molecular mechanisms by which HSP60 regulates the TLR4/MyD88/NF-*κ*B signaling pathway need to be further investigated.

IL-1*β* plays a pivotal role in the initiation of gout flare, but production and release of IL-1*β* is a multistep process. MSU crystals can activate the NLRP3 inflammasome, leading to activation of caspase 1, which enzymatically processes pro-IL-1*β* to bioactive IL-1*β*. IL-1*β* signaling initiates the production and secretion of proinflammatory mediators. Most studies support that MSU crystals can induce mitochondrial dysfunction, which is closely associated with the activation of the NLRP3 inflammasome. According to our data, HSP60 accelerated the expression of NLRP3 at transcription and protein levels once macrophages were activated by MSU crystals. A previous study reported that in N9 cells, HSP60 overexpression could affect p65 nuclear translocation and regulate NLRP3 expression [16]. In our study, HSP60 overexpression almost did not impact p65 nuclear translocation or the expression of Caspase1 p20 and IL-1*β* without MSU treatment. Perhaps in different cell lines, HSP60 functions somewhat differently. When macrophages were stimulated by MSU crystals, there was a large amount of HSP60 protein accumulated in the mitochondria. HSP60 can influence mitochondrial ROS induced by MSU crystals. The antioxidant defense system in mitochondria is very important for the maintenance of mitochondrial ROS. When THP-1-derived macrophages were treated with MSU crystals, the activity of SOD, CAT, and GSH-PX was significantly decreased. When the expression of HSP60 was inhibited in the MSU crystal-induced macrophages, the activity of these antioxidant enzymes is greatly enhanced, implying that HSP60 has the function of regulating oxidation balance. HSP60 also affects the production of mitochondrial ATP and the content of mitochondrial DNA. Further research should be performed to elucidate the molecular mechanisms of HSP60 involvement in the regulation of mitochondrial dysfunction.

To further explore the effect of HSP60 on MSU crystal-induced inflammation in vivo, we established mouse models of gout arthritis induced by MSU crystals. This process triggers a series of inflammatory reactions, similar to acute gout arthritis [14]. Vivo-morpholino can be injected directly into interested regions to achieve effective local delivery, so we directly injected it into the joint or abdominal cavity to suppress HSP60 expression. In MSU crystals injected into the foot pad and ankle joint, HSP60 knockdown relieved joint swelling and leukocyte infiltration to the inflamed tissues. MPO is a surrogate marker for granulocyte infiltration, and we also noticed a significant decrease in MPO activity in HSP60 MO injection foot pads compared to MSU crystals+Ctrl-MO group. This study demonstrated that the inhibition of HSP60 expression can alleviate the severity of arthritis induced by MSU crystals, which may provide a new therapeutic target for the treatment of gout arthritis.

**Figure 7:**
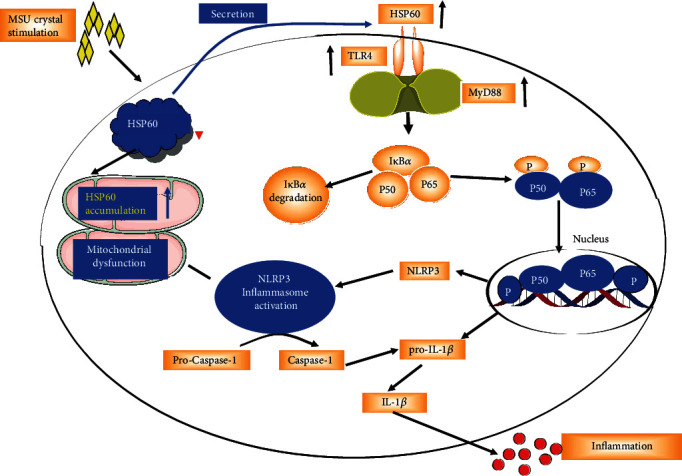
Schema of the signaling pathway involved in HSP60-mediated NLRP3 inflammasome activation and subsequent IL-1*β* production. After MSU crystal stimulation, the secretion of HSP60 to the extracellular level is greatly increased, which activates the signaling pathway and promotes the transcription of IL-1*β* and NLRP3. MSU crystal treatment also leads to a large amount of HSP60 aggregation in mitochondria, causing mitochondrial dysfunction, thereby activating NLRP3 inflammasome and promoting IL-1*β* precursor processing and ultimately leading to IL-1*β* release and inflammatory response.

Taken together, our study proposes that HSP60 activates the TLR4/MyD88/NF-*κ*B signaling pathway and induces mitochondrial damage to trigger the activation of NLRP3 inflammasome (Figure 7), thereby exerting an effect akin to promoting gout arthritis. It may be possible to target HSP60 for gout arthritis therapy in the future.

## Figures and Tables

**Table 1:** Characteristics of gout patients and healthy controls.

Items	AG	IG	HC
Case number	33	31	43
Gender	Male	Male	Male
Age years	46.5 ± 7.8	43.1 ± 11.5	45.2 ± 12.5
Disease duration (years)	4.6 ± 2.7^b^	7.5 ± 3.9	NA
BMI (kg/m^2^)	26.87 ± 4.21^a^	27.13 ± 4.63^a^	24.58 ± 3.58
WBC (×10^9^/L)	8.74 ± 2.47^a^	7.62 ± 2.03^a^	5.72 ± 2.38
CRP (mg/L)	48.29 ± 27.11^a,b^	2.89 ± 2.45^a^	2.14 ± 1.87
Neu (×10^9^/L)	5.26 ± 1.93^a,b^	3.98 ± 1.53	3.87 ± 1.26
Uric acid (*μ*mol/L)	507.33 ± 108.41^a^	482.19 ± 96.74^a^	213.17 ± 71.19

HC: healthy control; AG: acute gout; IG: intercritical gout; BMI: body mass index; WBC: white blood cell; CRP: C-reaction protein; UA: blood uric acid; Neu: neutrophil granulocyte absolute value; UA: blood uric acid concentration. ^a^*p* < 0.05 (in comparison with HC group); ^b^*p* < 0.05 (in comparison with the IG group).

**Table 2:** The primers used for quantitative PCR.

Items	Forward sequence (5′–3′)	Reverse sequence (5′–3′)
Human IL-1*β*	ATGGCTTATTACAGTGGC	AACCAGCATCTTCCTCAG
Human IL-6	CCTTAGCCCTGGAACTGC	AAGGCAACTGGACCGAAG
Human TNF-*α*	CGAGTCTGGGCAGGTCTA	GGTTTCGAAGTGGTGGTC
Human COX-2	CTCAGACGCTCAGGAAAT	AGTTGAAGATTAGTCCGC
Human iNOS	TTCAGTATCACAACCTCAGCAAG	TGGACCTGCAAGTTAAAATCCC
Human NLRP3	GATCTTCGCTGCGATCAACAG	CGTGCATTATCTGAACCCCAC
Human HSP60	ATGCTTCGGTTACCCACAGTC	AGCCCGAGTGAGATGAGGAG
Human *β*-actin	CTCCTCCTGAGCGCAAGTACTC	CTGCTTGCTGATCCACATCTG
Cytochrome *c* oxidase I for mouse mtDNA	GCCCCAGATATAGCATTCCC	GTTCATCCTGTTCCTGCTCC
18S ribosomal RNA for mouse nDNA	TAGAGGGACAAGTGGCGTTC	CGCTGAGCCAGTCAGTGT

## Data Availability

The [DATA TYPE] data used to support the findings of this study are included within the article.

## Supplementary Materials

Supplementary 1 Supplementary Figure 1: HSP60 expression, secretion, and localization were affected by MSU crystals in RAW264.7 cells. (a–c) RAW264.7 cells were primed with LPS (100 ng/ml) for 1 h and then treated with different concentrations of MSU suspension for 12 h. (a) Influence of MSU crystals on HSP60 secretion to the extracellular medium was analyzed by ELISA. (b) Representative images of double-labeling HSP60 and mitochondrial marker (MitoTracker), quantification of fluorescence intensity of HSP60 protein. Blue shows nuclei staining with DAPI. Scale bar: 20 *μ*m. Each experiment had six fields of view. Image J software was used to quantify the immunofluorescence staining of HSP60. (c) Western blot analysis was used to detect HSP60 protein levels in the mitochondrial and cytoplasmic extracts. HSP60 protein level in the mitochondria was normalized to VDCA1 and then averaged. HSP60 protein level in the cytoplasm was quantified by normalization of their densitometry to *β*-actin. ∗In comparison with the absence of MSU crystal treatment and # in comparison with 50 *μ*g/ml MSU crystal treatment.

Supplementary 2 Supplementary Figure 2: MSU crystal exposure increased HSP60 mRNA level and HSP60 secretion in a time-dependent manner. (a, b) RAW264.7 cells were primed with LPS (100 ng/ml) for 1 h and then treated with MSU crystals (100 *μ*g/ml) for different time point. (a) The relative mRNA expression of HSP60 was tested through RT-PCR. (b) HSP60 secretion was analyzed by ELISA. ∗*p* < 0.05, in comparison with the absence of MSU crystal treatment.

Supplementary 3 Supplementary Figure 3: TLR4 inhibitor (CLI-095) reversed the enhanced p-P65 and p-P50 proteins because of HSP60 overexpression in MSU crystal-induced RAW264.7 cells. After RAW264.7 cells were transfected with pcDNA3.1-HSP60 plasmid or pcDNA3.1 vector for 36 h, cells were treated with 0 or 5 *μ*M CLI-095 (TLR4 inhibit, Invitrogen) for 2 h and then primed with LPS for 1 h prior to MSU crystal stimulation (50 *μ*g/ml, MSU treatment 12 h). Experiments were repeated at least three times and data are shown as mean ± SEM. ∗*p* < 0.05.

Supplementary 4 Supplementary Figure 4: the effect of HSP60 knockdown or overexpression on the mitochondrial ROS and mitochondrial membrane potential in RAW264.7 cells. (a, c) RAW264.7 cells were transfected with control siRNA or HSP60 siRNA for 48 h, primed with LPS (100 ng/ml) for 1 h, and then treated with MSU suspension (100 *μ*g/ml) for 12 h. (b, d) RAW264.7 cells were transfected with control vector or HSP60 vector for 36 h, primed with LPS (100 ng/ml) for 1 h, and then treated with MSU suspension (100 *μ*g/ml) for 12 h. (a) The effect of HSP60 knockdown on the mitochondrial ROS, representative images of MitoTracker green and MitoROS staining. Blue shows nuclei staining with Hoechst33342. Scale bar: 10 *μ*m. (b) The effect of HSP60 overexpression on the mitochondrial ROS, representative images of MitoTracker green and MitoROS staining. Blue shows nuclei staining with Hoechst33342. Scale bar: 10 *μ*m. (c) The effect of HSP60 knockdown on the mitochondrial membrane potential (MMP), cells were stained using JC-1 probe. Blue shows nuclei staining with Hoechst33342. Scale bar: 10 *μ*m. (d) The effect of HSP60 overexpression on the mitochondrial membrane potential (MMP), cells were stained using JC-1 probe. Blue shows nuclei staining with Hoechst33342. Scale bar: 10 *μ*m. For mtROS and MMP analysis, >50 individual cells were imaged per group from 3 culture dishes. Data are representative of mean ± SEM for three experiments. Each experiment had 10 fields of view. ∗*p* < 0.05.

Supplementary 5 Supplementary Figure 5: MitoTEMPO reversed the enhanced IL-1*β* release in the culture supernatants because of HSP60 overexpression in MSU crystal-induced THP-1-derived macrophages. (a, c) THP-1-derived macrophages were transfected with control vector or HSP60 vector for 36 h, primed with LPS (100 ng/ml) for 1 h, and then treated with MSU suspension (50 *μ*g/ml) for 12 h. (a) The protein level of IL-1*β* was detected using western blot in the culture supernatants. (b) The protein level of IL-1*β* was detected using ELISA in the culture supernatants. Values are the mean ± SEM of 3 independent experiments. ∗*p* < 0.05.

Supplementary 6 Supplementary Figure 6: HSP60 downregulation decreased the protein levels of MPO, iNOS, and COX-2 in the foot pad tissue. Western blot was used to detect protein extracts from foot pad tissues, densitometry analysis of I*κ*B*α*, p-P50, and p-P65 protein. The data represent the mean ± SEM for three experiments. #Significantly different from the absence of MSU crystals injection mice and ∗ significantly different from MSU crystals and ctrl MO injection mice.
