# Supplementary figures and images for: HSP60 Regulates Monosodium Urate Crystal-Induced Inflammation by Activating the TLR4-NF-κB-MyD88 Signaling Pathway and Disrupting Mitochondrial Function
Source: Oxid Med Cell Longev. 2020 Dec 31;2020:8706898. doi: 10.1155/2020/8706898 (PMC7791970; doi:10.1155/2020/8706898)

# Supplementary Figure 1

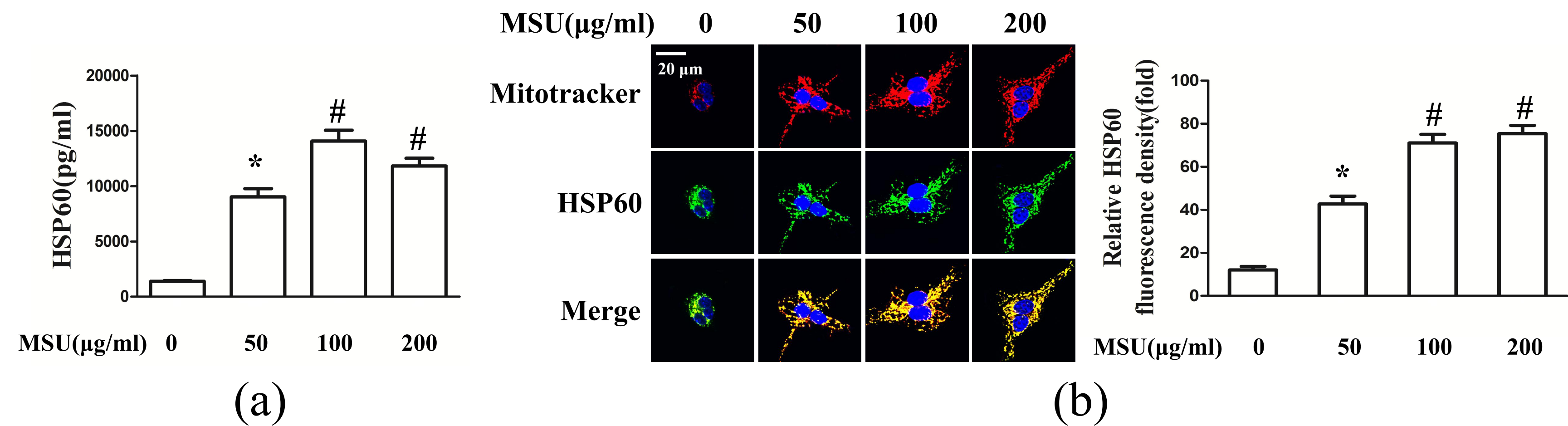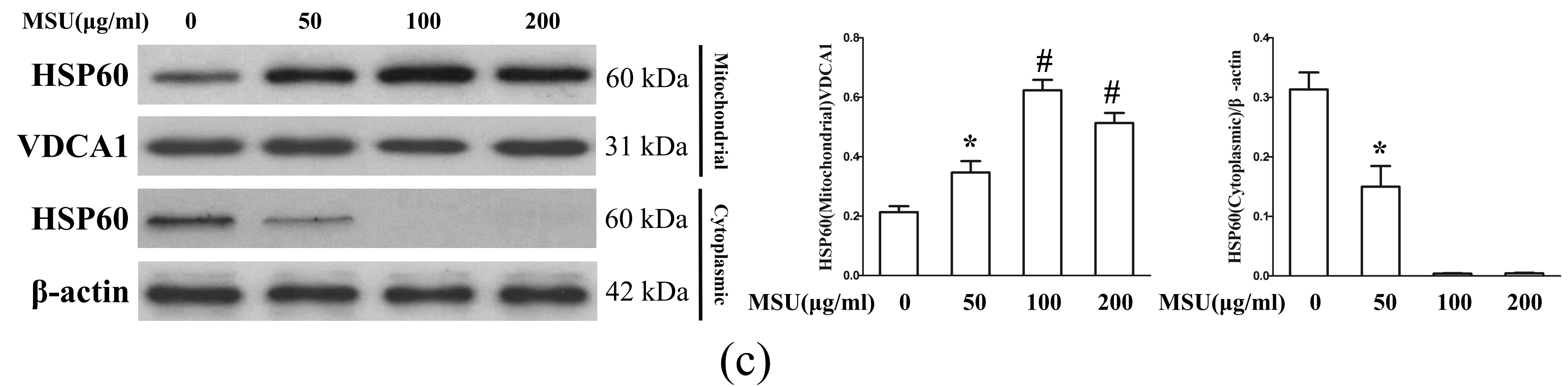

Supplement: Supplementary 1 — Supplementary Figure 1: HSP60 expression, secretion, and localization were affected by MSU crystals in RAW264.7 cells. (a–c) RAW264.7 cells were primed with LPS (100 ng/ml) for 1 h and then treated with different concentrations of MSU suspension for 12 h. (a) Influence of MSU crystals on HSP60 secretion to the extracellular medium was analyzed by ELISA. (b) Representative images of double-labeling HSP60 and mitochondrial marker (MitoTracker), quantification of fluorescence intensity of HSP60 protein. Blue shows nuclei staining with DAPI. Scale bar: 20 μm. Each experiment had six fields of view. Image J software was used to quantify the immunofluorescence staining of HSP60. (c) Western blot analysis was used to detect HSP60 protein levels in the mitochondrial and cytoplasmic extracts. HSP60 protein level in the mitochondria was normalized to VDCA1 and then averaged. HSP60 protein level in the cytoplasm was quantified by normalization of their densitometry to β-actin. ∗In comparison with the absence of MSU crystal treatment and # in comparison with 50 μg/ml MSU crystal treatment. [file 8706898.f1.pdf]

# Supplementary Figure 2

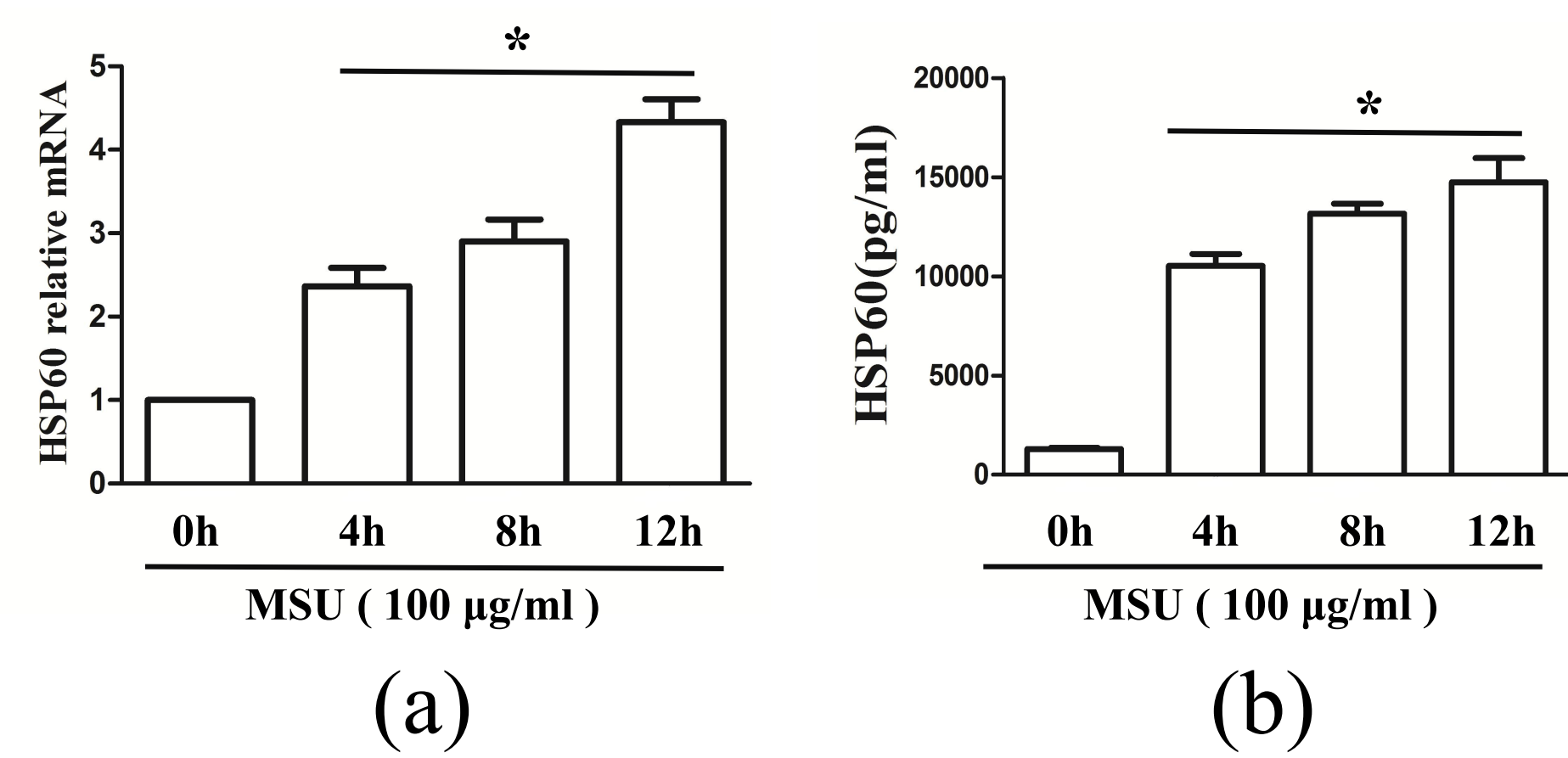

Supplement: Supplementary 2 — Supplementary Figure 2: MSU crystal exposure increased HSP60 mRNA level and HSP60 secretion in a time-dependent manner. (a, b) RAW264.7 cells were primed with LPS (100 ng/ml) for 1 h and then treated with MSU crystals (100 μg/ml) for different time point. (a) The relative mRNA expression of HSP60 was tested through RT-PCR. (b) HSP60 secretion was analyzed by ELISA. ∗p < 0.05, in comparison with the absence of MSU crystal treatment. [file 8706898.f2.pdf]

Supplementary Figure 3

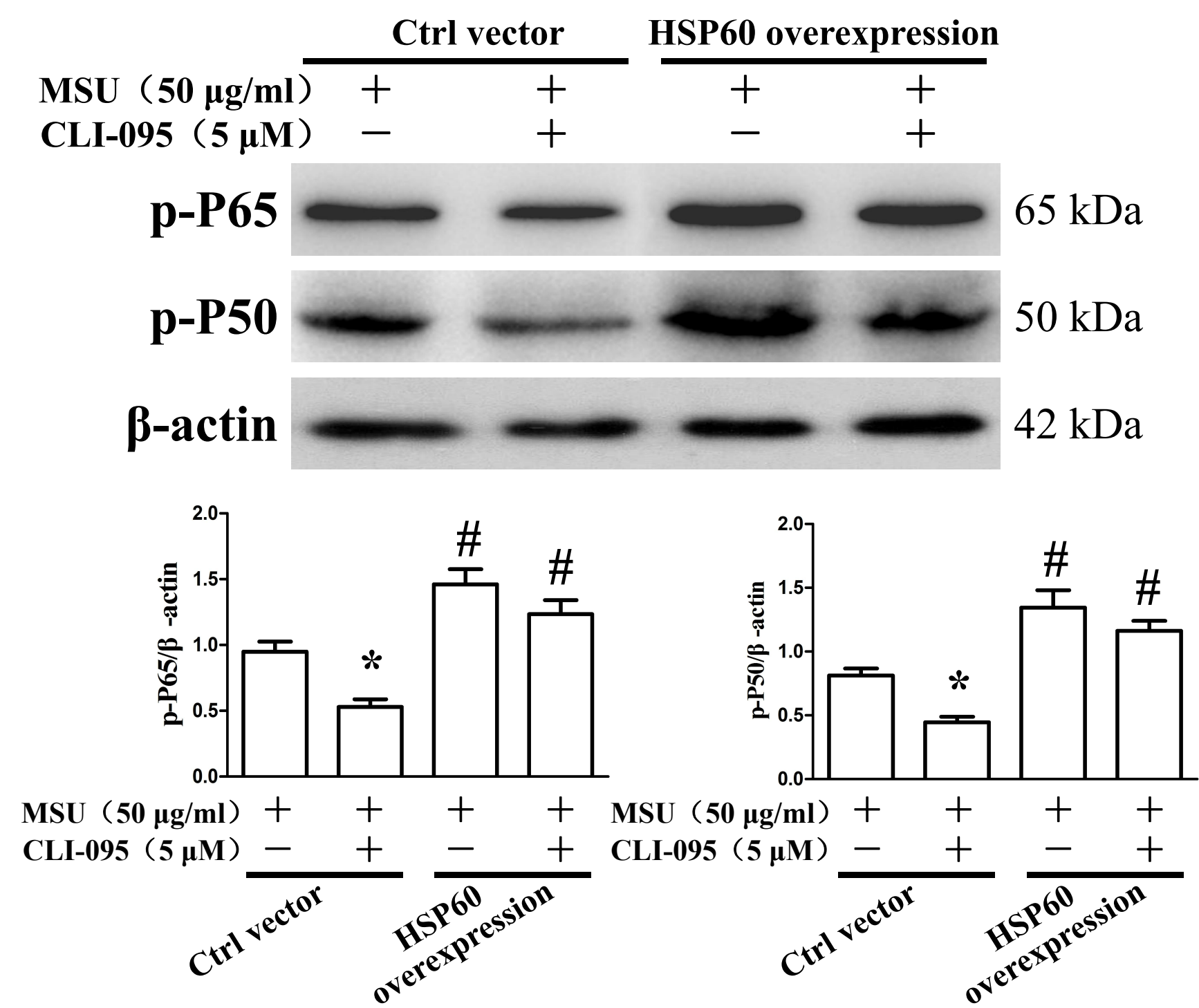

Supplement: Supplementary 3 — Supplementary Figure 3: TLR4 inhibitor (CLI-095) reversed the enhanced p-P65 and p-P50 proteins because of HSP60 overexpression in MSU crystal-induced RAW264.7 cells. After RAW264.7 cells were transfected with pcDNA3.1-HSP60 plasmid or pcDNA3.1 vector for 36 h, cells were treated with 0 or 5 μM CLI-095 (TLR4 inhibit, Invitrogen) for 2 h and then primed with LPS for 1 h prior to MSU crystal stimulation (50 μg/ml, MSU treatment 12 h). Experiments were repeated at least three times and data are shown as mean ± SEM. ∗p < 0.05. [file 8706898.f3.pdf]

Supplementary Figure 4

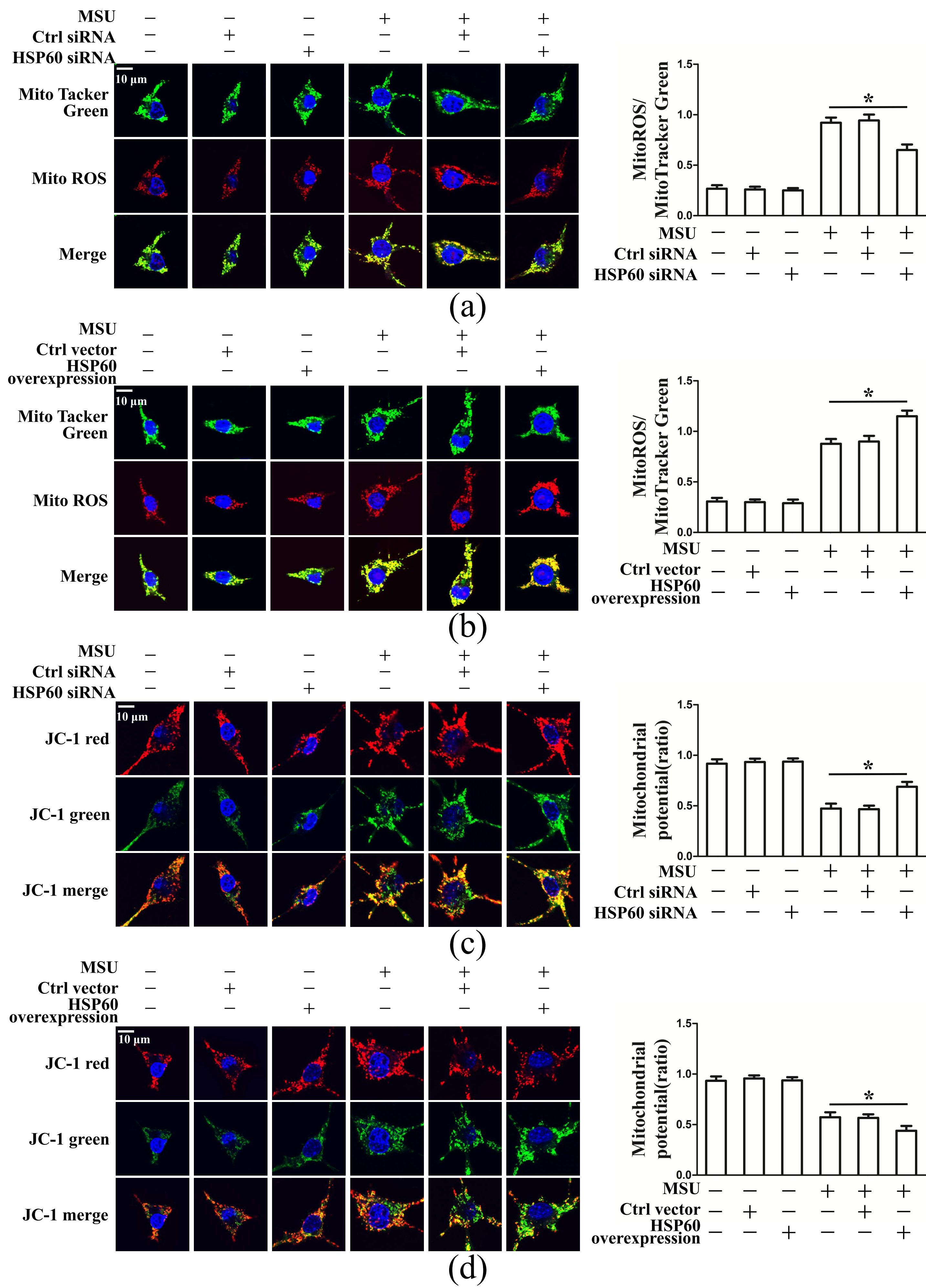

Supplement: Supplementary 4 — Supplementary Figure 4: the effect of HSP60 knockdown or overexpression on the mitochondrial ROS and mitochondrial membrane potential in RAW264.7 cells. (a, c) RAW264.7 cells were transfected with control siRNA or HSP60 siRNA for 48 h, primed with LPS (100 ng/ml) for 1 h, and then treated with MSU suspension (100 μg/ml) for 12 h. (b, d) RAW264.7 cells were transfected with control vector or HSP60 vector for 36 h, primed with LPS (100 ng/ml) for 1 h, and then treated with MSU suspension (100 μg/ml) for 12 h. (a) The effect of HSP60 knockdown on the mitochondrial ROS, representative images of MitoTracker green and MitoROS staining. Blue shows nuclei staining with Hoechst33342. Scale bar: 10 μm. (b) The effect of HSP60 overexpression on the mitochondrial ROS, representative images of MitoTracker green and MitoROS staining. Blue shows nuclei staining with Hoechst33342. Scale bar: 10 μm. (c) The effect of HSP60 knockdown on the mitochondrial membrane potential (MMP), cells were stained using JC-1 probe. Blue shows nuclei staining with Hoechst33342. Scale bar: 10 μm. (d) The effect of HSP60 overexpression on the mitochondrial membrane potential (MMP), cells were stained using JC-1 probe. Blue shows nuclei staining with Hoechst33342. Scale bar: 10 μm. For mtROS and MMP analysis, >50 individual cells were imaged per group from 3 culture dishes. Data are representative of mean ± SEM for three experiments. Each experiment had 10 fields of view. ∗p < 0.05. [file 8706898.f4.pdf]

Supplementary Figure 5

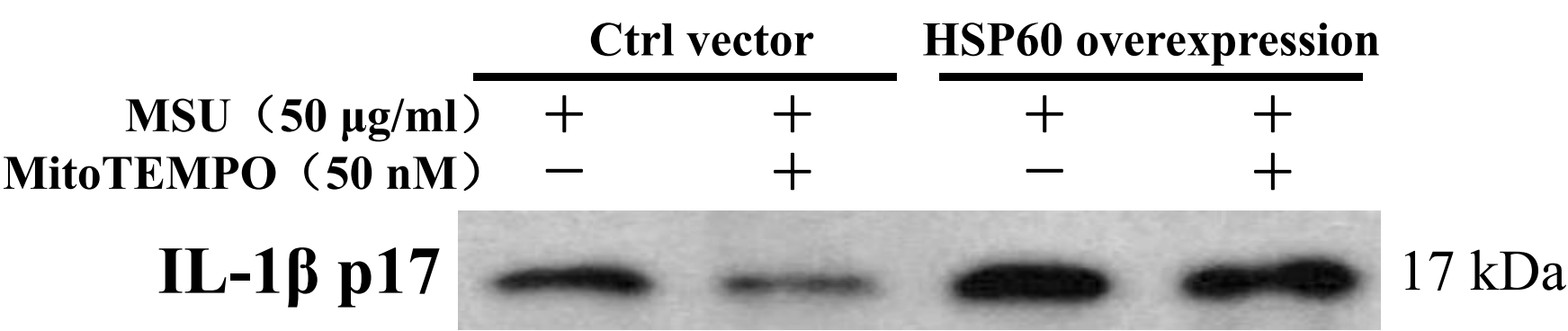

(a)

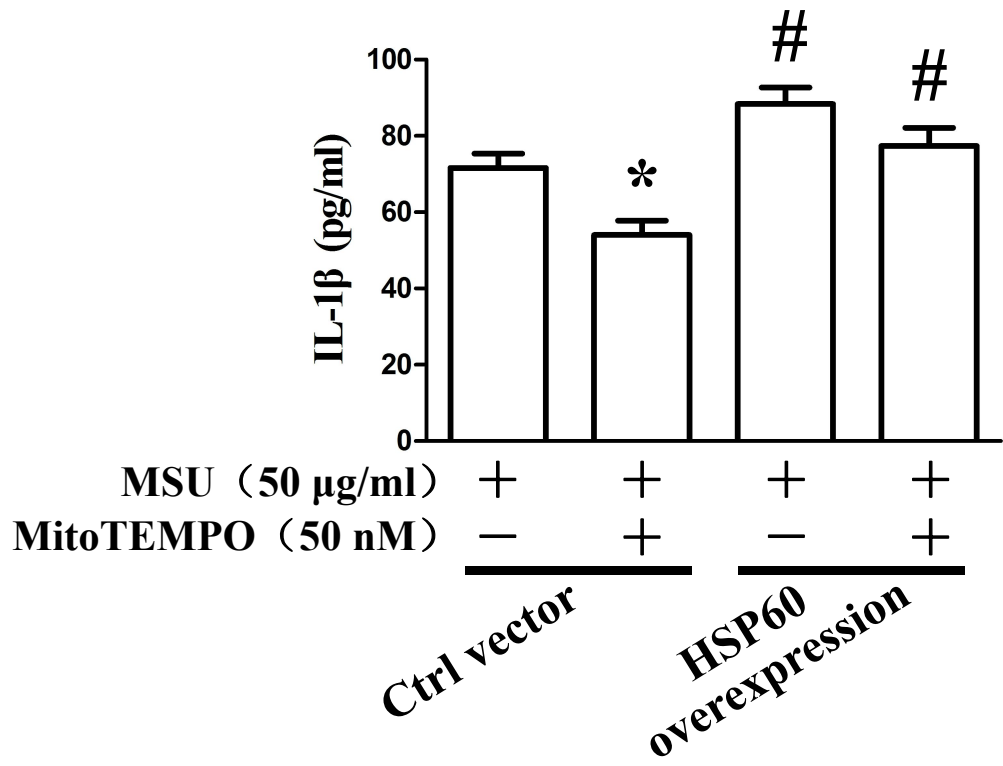

(b)

Supplement: Supplementary 5 — Supplementary Figure 5: MitoTEMPO reversed the enhanced IL-1β release in the culture supernatants because of HSP60 overexpression in MSU crystal-induced THP-1-derived macrophages. (a, c) THP-1-derived macrophages were transfected with control vector or HSP60 vector for 36 h, primed with LPS (100 ng/ml) for 1 h, and then treated with MSU suspension (50 μg/ml) for 12 h. (a) The protein level of IL-1β was detected using western blot in the culture supernatants. (b) The protein level of IL-1β was detected using ELISA in the culture supernatants. Values are the mean ± SEM of 3 independent experiments. ∗p < 0.05. [file 8706898.f5.pdf]

# Supplementary Figure 6

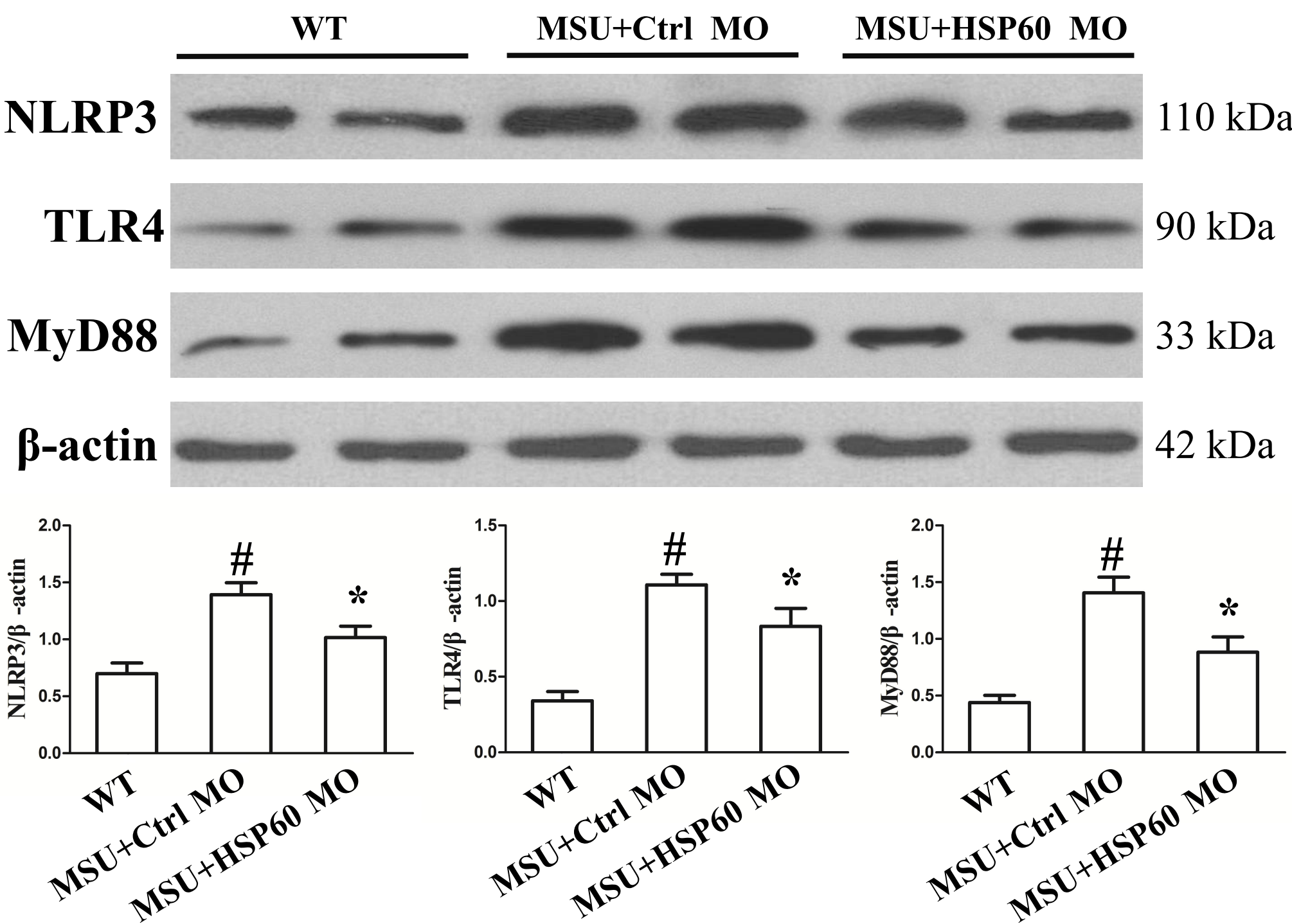

Supplement: Supplementary 6 — Supplementary Figure 6: HSP60 downregulation decreased the protein levels of MPO, iNOS, and COX-2 in the foot pad tissue. Western blot was used to detect protein extracts from foot pad tissues, densitometry analysis of IκBα, p-P50, and p-P65 protein. The data represent the mean ± SEM for three experiments. #Significantly different from the absence of MSU crystals injection mice and ∗ significantly different from MSU crystals and ctrl MO injection mice. [file 8706898.f6.pdf]
